# Supplementary material for: The psychophysics of bouncing: Perceptual constraints, physical constraints, animacy, and phenomenal causality
Source: PLoS One. 2023 Aug 18;18(8):e0285448. doi: 10.1371/journal.pone.0285448 (PMC10437946; doi:10.1371/journal.pone.0285448)
Supplement: Attachment — Submitted filename: PONE-D-22-33138_review.pdf [file pone.0285448.s001.pdf]

The psychophysics of bouncing: perceptual constraints, physical constraints, animacy, and phenomenal causality

This study reports 4 experiments investigating which visual parameters (kinematics, presence or not of a bouncing surface) produce physical or animacy (or neither) interpretations of a bouncing motion. Although I found most of the experimental manipulations relevant and I may agree with the theoretical framing used for interpreting the findings, I think as it stands, the statistical analyses do not do the readers any favors or do the data justice in order to help draw out the essence of the data. That is why the bulk of my report will focus on these analyses, and I recommend a major revision.

First, I must confess that I had great difficulty in following the exposure of results starting with the Box and Whisker plots used in Figure 5 (Expt1) and 6 (Expt2). By the way, the authors abandoned such Figures in the subsequent experiments. Why use these nonparametric plots to illustrate results from parametric Generalized Linear Mixed Models (GLMM, here, with a logit link function for the logistic regressions)? GLMM typically illustrate Fixed and random effects in a very different way than in the manuscript. I give some illustrations in Appendices 1-3 (after all my comments), for Experiment 2 data that I downloaded from the link provided in the manuscript. When predictors are treated as "factors" then, the figures look like ANOVA figures (see Appendix 1 and 3). Moreover, in this case the random effects plotted by participant should be added to the figures as separate lines in order to better visualize the factors effects at the individual, which cannot be visualized in the kind of plots actually used by the authors. In contrast, if some experimental factors are treated as continuous variables (covariates), the predictor effect may be visualized using logistic curves, or with linear functions after logit transform of the dependent variable (see Appendix 2). Examples of these figures can be found in: https://gamlj.github.io/glmmixed_example1.html#The_research_design

Moreover, the reason for using GLMM (sometimes also called MLM, multi level models) instead to ANOVA is unclear/unjustified. The authors just refer Jaeger (2008, doi: 10.1016/j.jml.2007.11.007) paper who promoted using Logit Mixed Models instead of ANOVAs over proportions of categorical outcomes. Among others, one argument was "*even ANOVAs over arcsine-square-root transformed proportions of categorical outcomes* (see below*) can lead to spurious null results and spurious significances*. These spurious results go beyond the normal chance of Type I and Type II errors. " (Jaeger, 2008, p.435). However, recent papers clearly challenge Jaeger (2008) conclusions with simulation studies that plead for the use of repeated-measures ANOVA with Huynh-Feldt-correction (especially when the sphericity assumption is violated), rather than multi-level models, when the sample size is rather small and the number of measurement occasions is large (Haverkamp & Beauducel, 2017, DOI: 10.3389/fpsyg.2017.01841). Likewise, Luepsen (2021, DOI: 10.1080/03610918.2020.1869983) simulation study showed that for binary variables, "Depending on the design and on the model to be checked, in most cases the parametric F-test with adjustment, the multivariate test or Koch's method are advised", as compared to GLMM and GEE (Generalized Estimating Equations).

What's more, the way authors use GLMM is unconventional. The standard approach is first to test the intercept only model (Null model), to check if there is substantial evidence of clustering in the data to justify a multilevel approach. In fact, this does not seem to be the case in Experiment 2 for the "physical" response dependent variable (see the Tables below) as suggested by the ICC (intraclass correlation) values that are smaller than 0.05 which is a usual cut-off value[1] above which to go on with GLMM approach (when ICC > 0.05). ICC quantifies the proportion of variance explained by a grouping/random factor in multilevel data, and corresponds to the R^2 conditional value in the null model.

[1] Along the lines of Hox (2010) suggesting "using .05, .10, and .15 as small, medium, and large values for the intraclass correlation in general cases » (p. 244)

Null model for Expt2 « physical responses »

Info	Value	Comment
Model Type	Logistic	Model for binary y
Call	glm	physical ~ 1 + (1 | participant)
Link function	Logit	Log of the odd of y=1 over y=0
Direction	P(y=1)/P(y=0)	P(physical = 1) / P(physical = 0)
Distribution	Binomial	Dichotomous event distribution of y
LogLikel.	-1876.8896	Unconditional Log-Likelihood
-2*LogLikel.	3753.7792	Unconditional absolute deviance
Deviance	3697.8981	Conditional relative deviance
R-squared	0.0000	Marginal
R-squared	0.0303	Conditional
AIC	3757.7800	Less is better
BIC	3769.7103	Less is better
Residual DF	2878.0000	
Chi-squared/DF	0.9835	Overdispersion indicator
Converged	yes	
Optimizer	bobyqa	

Random Components (Null model)

Groups	Name	SD	Variance	ICC
participant	(Intercept)	0.321	0.103	0.0303
Residuals		1.000	1.000	.

Note. Number of Obs: 2880 , groups: participant 30

It is neither the case for the "animated" responses :

Random Components (Null model) – « animated » response

Groups	Name	SD	Variance	ICC
participant	(Intercept)	0.363	0.132	0.0385
Residuals		1.000	1.000	.

Note. Number of Obs: 2880 , groups: participant 30

Only the null model for « Other » responses shows some clustering in the data. But these are not the responses of interest at the core of the study.

Random Components (Null model) – « Other » response

Groups	Name	SD	Variance	ICC
participant	(Intercept)	1.55	2.39	0.421
Residuals		1.00	1.00	.

Note. Number of Obs: 2880 , groups: participant 30

Likewise, instead of listing all the comparisons and associated odds ratio (OR), these OR could be visualized in a figure (see https://strengejacke.github.io/sjPlot/articles/plot_model_estimates.html)

with the interpretation summarized in a few sentences without the *p* values (these could be provided in Supplementary Material). The idea, here, is to facilitate the reading of results that are presented in a rather choppy way for the moment. Jtools is perfect for summarizing and visualizing regression models (https://jtools.jacob-long.com/articles/summ.html)

Furthermore, I don't understand why interaction terms were not introduction in the GLMM models of the authors. Actually, one should start with a null model to test if the GLMM approach is justified (by looking at ICC of the null model) and if so, then increase the complexity of the model up to the point of including interactions. Then redundant predictors need to be identified (looking at correlations) and deleted from the model, until a fair tradeoff between explained variance and complexity of the model is met. This is not at all the way the authors use GLMM. For examples of *Logistic Mixed Effects Model with Interaction Term* and *Logistic Mixed Effects Model with Three-Way Interaction* (with lme4, glmer):

- https://strengejacke.github.io/ggeffects/articles/practical_logisticmixedmodel.html
- Hox (2010) *Multilevel Analysis Techniques and Applications* Second Edition. Routledge
- See also Mike Crowson tutorials: https://www.youtube.com/watch?v=8r9bUKUVecc

Instead the authors chose to perform multiple GLMM sub-analyses, that are equivalent to performing *t* tests (actually the authors used z scores and odds ratio) on multiple paired comparisons to decompose an ANOVA interaction, without showing first that the interaction is significant! Also, the pairwise analysis is not homogenous across the experiments. For example, the authors performed paired comparisons between adjacent delay levels (without controlling for multiple testing!) in Experiment 2, but not for the a and C levels in Experiment 1.

In addition, stimuli could have been treated as random factors[2] (see Judd et al 2012 DOI: 10.1037/a0028347) and interaction between the random effects studied, from the start in Expt1 and 2. Thereby level of "C" or "delay" factors would be considered just samples of possible stimuli out of a larger variation in levels of stimuli. Unfortunately, authors waited Expt3 and 4 before touching on the possibilities offered by this approach but without reporting/discussing the variances nor the ICC values nor the correlations between random effects the added value of these analyses (as compared to not including stimuli as random effects) is simply lost.

Finally, why not using random intercept and slope models, that would possibly increase the explanatory power of GLMM, as suggested by Appendix 4 for an example for their Expt2. Likelihood Ratio Test statistic (LRT) is also useful to test if larger models provide significant improvement over reduced models (see Roback & Legler, 2021, https://bookdown.org/roback/bookdown-BeyondMLR/), and also to analyze the variance components of the model. Regarding model comparisons, see : Raudenbush, S. W., & Bryk, A. S. (2002). *Hierarchical linear models: Applications and data analysis methods* (2nd ed.). Newbury Park: Sage.

In sum, given all the above issues, instead of using a GLMM approach I would rather suggest that the authors use Huynh-Feldt corrected ANOVAs, and interpret the parameters of the associated psychometric functions (instead of testing multiple pairwise comparisons to each stimulus level of the psychometric function), starting with an omnibus ANOVA on response proportion using the different response categories are levels of a factor, as in the Appendix 5 example for their Expt2. Then, onmnibus ANOVAs may be run on each separate response type (provided that response category interacted with the other within-subject factors in the initial ANOVA), followed by sub-ANOVAs for decomposing complex interactions. Additionally, the authors could either make an ANOVA on the

[2] The idea of treating stimuli as random factors/effects is to test if the results are generalizable not only to other samples of participants, but also to other samples of stimuli. In other words, participants are not the only random effect: this is also the case for the factor studied. By considering the factor studied as a fixed effect, we do not take into account the sampling bias associated with the choice of stimuli (especially for delay or C in the present study): if we had used another sample of stimuli, the results would have been slightly different. The difference between the estimated means of the experimental conditions is thus due not only to the manipulated factor, but also to this bias. By not removing the sampling variance from the estimate, the effect of the factor is overestimated. See "Subjects by Stimuli random effects in Mixed Models" https://gamlj.github.io/mixed_example3.html

psychometric lapse rate, guess rate, PSE, and JND parameters (see Kingdom & Prins, 2010, *Psychophysics: A practical introduction* book) data for each individual, or describe the above mentioned ANOVA interactions on the basis of the psychometric parameters computed from the mean response proportions (see examples below) rather than use pairwise comparisons. Note that PSE (transition point between response categories) and JND parameters are the main parameters of interest.

For example, according to the Figure below, the interaction between C and Delay ($F(5, 145)= 11.13$, $p < 0.0001$, H-F epsilon = 0.83) on the Proportion of "physical responses" in Experiment 2, reflects similar transition point (about a 68 ms delay) from physical to animated&other responses for both C levels, but a greater change in response for C = 0.75 compared to C = 1.25 (see JND, lapse and guess parameters).

Note that the authors did not focus on these transition values (PSE), neither for delay in Expt 2, nor for C in Expt1, although they summarize the psychophysical effect of the experimental manipulations.

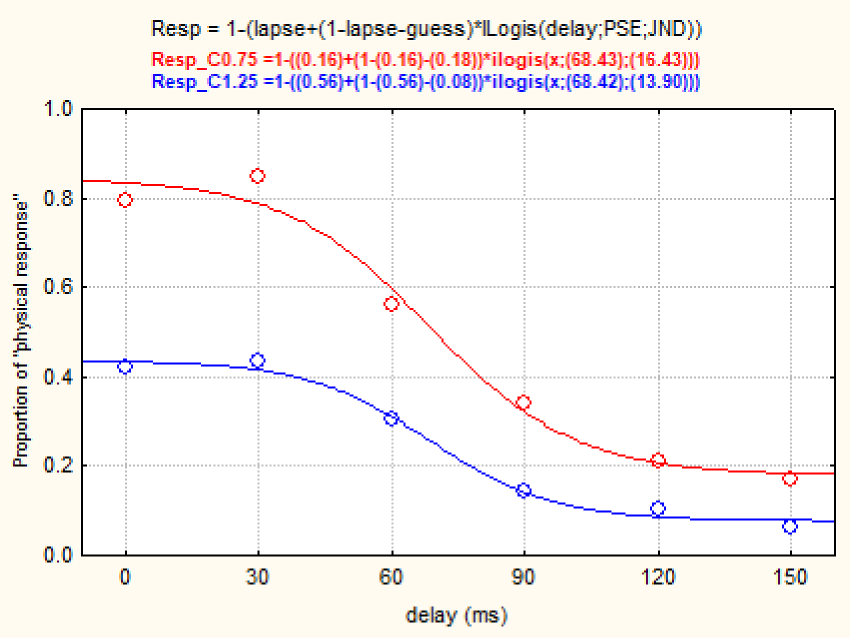

Likewise, **the multiple pairwise analyses of Experiment 1 also boil down to the logistic psychometric parameters whether using CDF or PDF**. For example, the interaction between C and Motion pattern on the Proportion of "physical responses" ($F(8, 232)= 8.14$, $p < 0.0001$, H-F epsilon = 0.832) illustrated below for the 3 bouncing cycles condition simply shows that the transition point from physical to animated&other responses is much earlier for the uniform acceleration motion pattern (about C= 0.63) as compared to the uniform acceleration motion pattern (about C= 0.81).

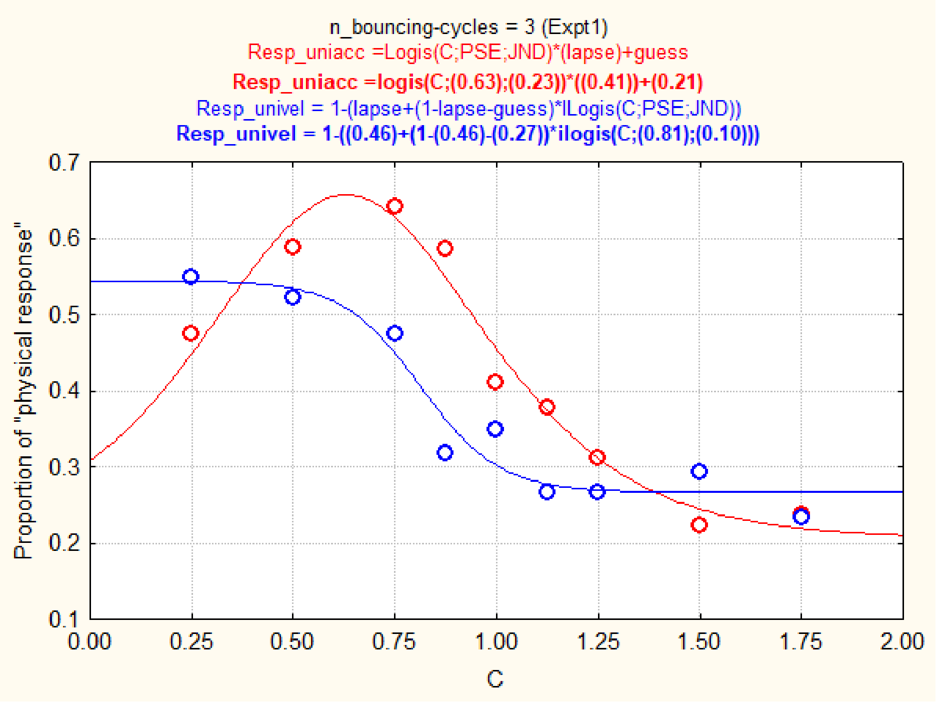

Miscellaneous

In Experiment 1, from the results for the "varying a" and the "varying C" animations (see Fig. 4), authors conclude that "*None of these animations gave rise to clear physical bounce or animated motion impressions*." In order to make the transition between Expt1 and 2 clearer, I suggest to move the exposure of these experimental manipulations in a Supplementary material section.

Likewise, I wonder if the analysis of "other" responses should be included in the main text. First, these responses were not analyzed in Experiment 2 (due to their low frequency). Second, this response category is not central for the discussion of the study. I suggest to move the data analysis for "other" responses in the Supplementary Material. As a consequence, it could therefore be removed from omnibus ANOVAs in the main text, if one starts with a "response category" factor with two levels (physical, animated) such as in the Appendix 6 example.

APPENDIX 1

Experiment 2, Sub-Part of manuscript Table 1 :

	Physical bounce		
	**Odds Ratio**	*z*	*p*
**30 vs 0 ms**	1.64	1.3	.19
**60 vs 30 ms**	0.27	-3.68	<.001*
**90 vs 60 ms**	0.47	-2.52	.012*
**120 vs 90 ms**	0.49	-2.23	.026*
**150 vs 120 ms**	0.55	-1.69	.09

* = $p < .05$

Note that the analyses below (using *Jamovi*) replicate exactly the results for the 90 vs 60 ms comparison and approximately for the other comparisons…

The 1/OR column was added because it corresponds to the Odds ratio reported in Expt 2, Table 1, of the manuscript. For example, the authors computed the odds ratio of the "physical" response for 90ms over 60 ms, which is 0.47, whereas here the counterpart odds ratio was used, that for 60ms delay over 90 ms, which is 1/0.47 = 2.13 (see column exp(B))

Generalized Mixed Models (Expt2), "physical" response as dependent variable, delay as a factor fixed effect, and participant as a random effect, ***for the "C = 0.75 and one bounce" data***

Glm call : physical ~ 1 + delay + (1 | participant)

Marginal R^2 = 0.202, conditional R^2 = 0.500

Effects Plots (*for the "C = 0.75 and one bounce" data*)

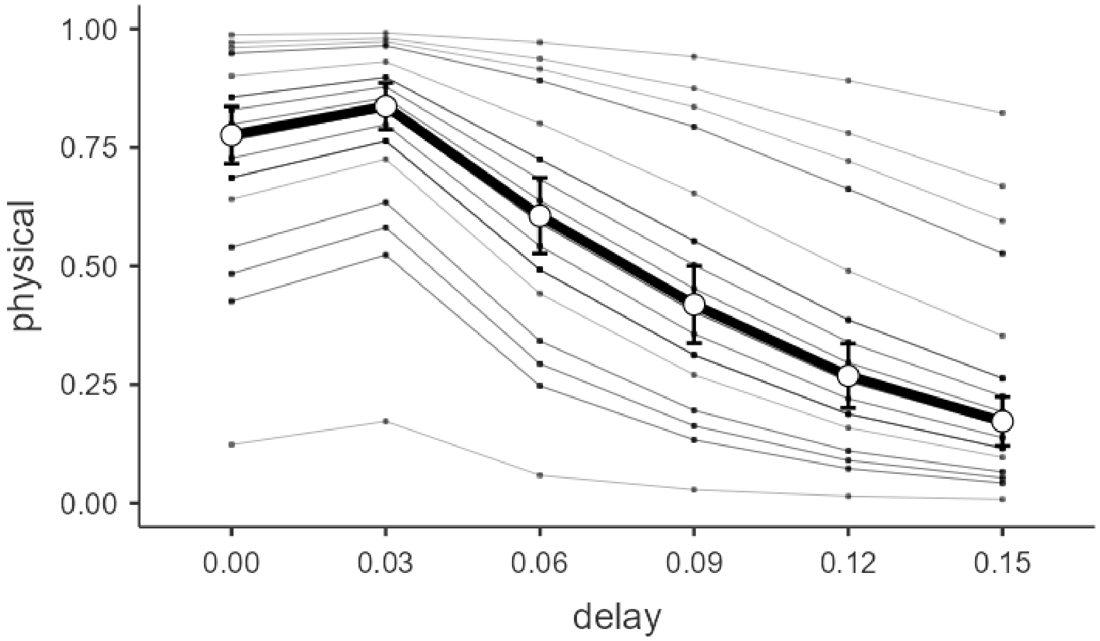

Note: Mean effect ±SE, and Random effects plotted by participant

Post Hoc Comparisons – « delay »

Comparison								
delay (s)		delay (s)	exp(B) = OR	SE	z	*p*	$p_{bonferroni}$	1/OR
**0.00**	**-**	**0.03**	**0.675**	0.227	**-1.17**	**0.242**	1.000	**1.48**
0.00	-	0.06	2.254	0.705	2.60	0.009	0.139	
0.00	-	0.09	4.813	1.526	4.96	< .001	< .001	
0.00	-	0.12	9.443	3.139	6.75	< .001	< .001	
0.00	-	0.15	16.639	5.901	7.93	< .001	< .001	
**0.03**	**-**	**0.06**	**3.339**	1.090	**3.69**	**< .001**	0.003	**0.30**
0.03	-	0.09	7.128	2.366	5.92	< .001	< .001	
0.03	-	0.12	13.986	4.859	7.59	< .001	< .001	
0.03	-	0.15	24.644	9.100	8.68	< .001	< .001	
**0.06**	**-**	**0.09**	**2.135**	0.642	**2.52**	**0.012**	0.175	**0.47**
0.06	-	0.12	4.189	1.318	4.55	< .001	< .001	
0.06	-	0.15	7.380	2.485	5.94	< .001	< .001	
**0.09**	**-**	**0.12**	**1.962**	0.612	**2.16**	**0.031**	0.461	**0.51**
0.09	-	0.15	3.457	1.148	3.73	< .001	0.003	
**0.12**	**-**	**0.15**	**1.762**	0.596	**1.67**	**0.094**	1.000	**0.57**

Generalized Mixed Models (Expt2), "physical" response as dependent variable, delay and C as covariates, bounce_cycles as factor

Model Info

Info	Value	Comment
Model Type	Logistic	Model for binary y
Call	glm	**physical ~ 1 + n_bouncing_cycles + delay + C + (1 | participant)**
Link function	Logit	Log of the odd of y=1 over y=0
Direction	P(y=1)/P(y=0)	P(physical = 1) / P(physical = 0)
Distribution	Binomial	Dichotomous event distribution of y
LogLikel.	-1455.190	Unconditional Log-Likelihood
-2*LogLikel.	2910.379	Unconditional absolute deviance
Deviance	2842.303	Conditional relative deviance
**R-squared**	**0.358**	**Marginal**
**R-squared**	**0.398**	**Conditional**
AIC	2920.380	Less is better
BIC	2950.207	Less is better
Residual DF	2875.000	
Chi-squared/DF	0.959	Overdispersion indicator
Converged	yes	
Optimizer	bobyqa	

Model Results

Fixed Effect Omnibus tests

	X^2	df	p
n_bouncing_cycles	63.2	1.00	< .001
delay	463.9	1.00	< .001
C	220.6	1.00	< .001

Fixed Effects Parameter Estimates

Names	Effect	Estimate	SE	exp(B)	z	*p*
(Intercept)	(Intercept)	-0.782	0.0985	0.4575	-7.94	< .001
n_bouncing_cycles1	3 - 1	-0.741	0.0932	0.4766	-7.95	< .001
delay	delay	-22.287	1.0347	2.09e-10	-21.54	< .001
C	C	-2.868	0.1931	0.0568	-14.85	< .001

Random Components

Groups	Name	SD	Variance	ICC
participant	(Intercept)	0.469	0.220	0.0626
Residuals		1.000	1.000	.

Note. Number of Obs: 2880 , groups: participant 30

APPENDIX 2

Generalized Mixed Models (Expt2), “physical” response, delay and C as covariates, n_bounce_cycles as factor

Model Info

Info	Value	Comment
Model Type	Logistic	Model for binary y
Call	glm	**physical ~ 1 + n_bouncing_cycles + delay + C + (1 | participant)**
Link function	Logit	Log of the odd of y=1 over y=0
Direction	P(y=1)/P(y=0)	P(physical = 1) / P(physical = 0)
Distribution	Binomial	Dichotomous event distribution of y
LogLikel.	-1455.190	Unconditional Log-Likelihood
-2*LogLikel.	2910.379	Unconditional absolute deviance
Deviance	2842.303	Conditional relative deviance
**R-squared**	**0.358**	**Marginal**
**R-squared**	**0.398**	**Conditional**
AIC	2920.380	Less is better
BIC	2950.207	Less is better
Residual DF	2875.000	
Chi-squared/DF	0.959	Overdispersion indicator
Converged	yes	
Optimizer	bobyqa	

Model Results

Fixed Effect Omnibus tests

	X^2	df	p
n_bouncing_cycles	63.2	1.00	< .001
delay	463.9	1.00	< .001
C	220.6	1.00	< .001

Fixed Effects Parameter Estimates

Names	Effect	Estimate	SE	exp(B)	z	*p*
(Intercept)	(Intercept)	-0.782	0.0985	0.4575	-7.94	< .001
n_bouncing_cycles1	3 - 1	-0.741	0.0932	0.4766	-7.95	< .001
delay	delay	-22.287	1.0347	2.09e-10	-21.54	< .001
C	C	-2.868	0.1931	0.0568	-14.85	< .001

Random Components

Groups	Name	SD	Variance	ICC
participant	(Intercept)	0.469	0.220	**0.0626**
Residuals		1.000	1.000	.

Note. Number of Obs: 2880 , groups: participant 30

Simple Effects

Simple effects of C : Omnibus Tests

Moderator levels			
**delay**	**X^2**	**df**	**p**
Mean-1-SD	221	1.00	< .001
Mean	221	1.00	< .001
Mean+1-SD	221	1.00	< .001

Simple effects of C : Parameter estimates

Moderator levels				95% Exp(B) Confidence Interval			
**delay**	**Estimate**	**SE**	**exp(B)**	**Lower**	**Upper**	**z**	**p**
Mean-1-SD	-2.87	0.193	0.0568	0.0389	0.0829	-14.9	< .001
Mean	-2.87	0.193	0.0568	0.0389	0.0829	-14.9	< .001
Mean+1-SD	-2.87	0.193	0.0568	0.0389	0.0829	-14.9	< .001

Note. No interaction involving the simple effects variables is present in the model. Simple effects are equivalent to main effects

Results Plots

n_bouncing_cycles = 1

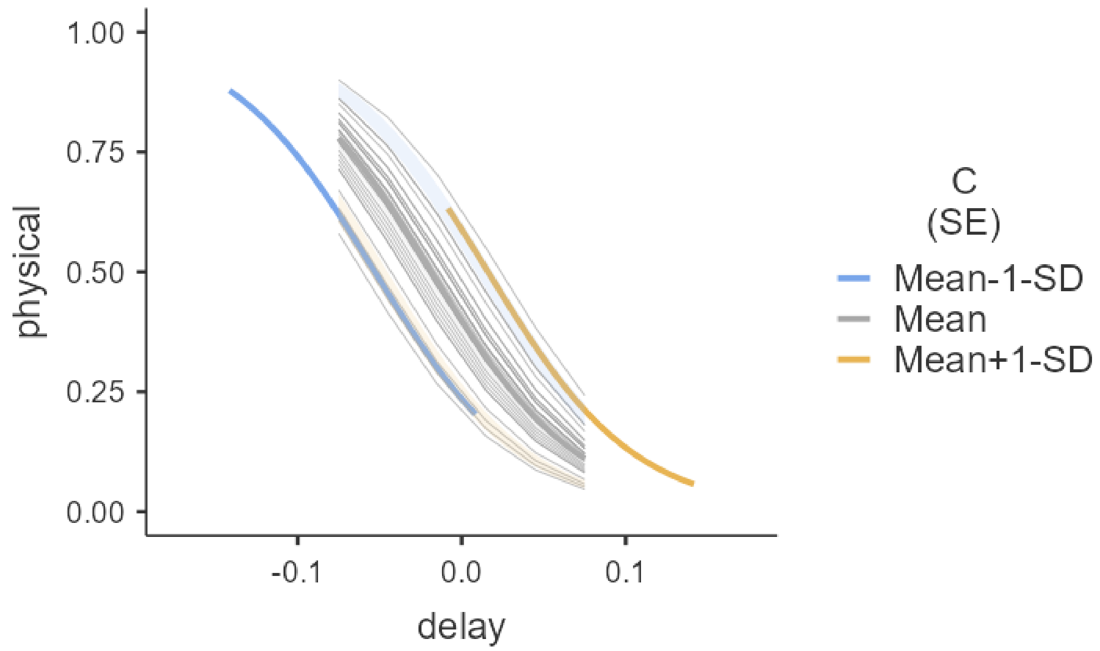

n_bouncing_cycles = 3

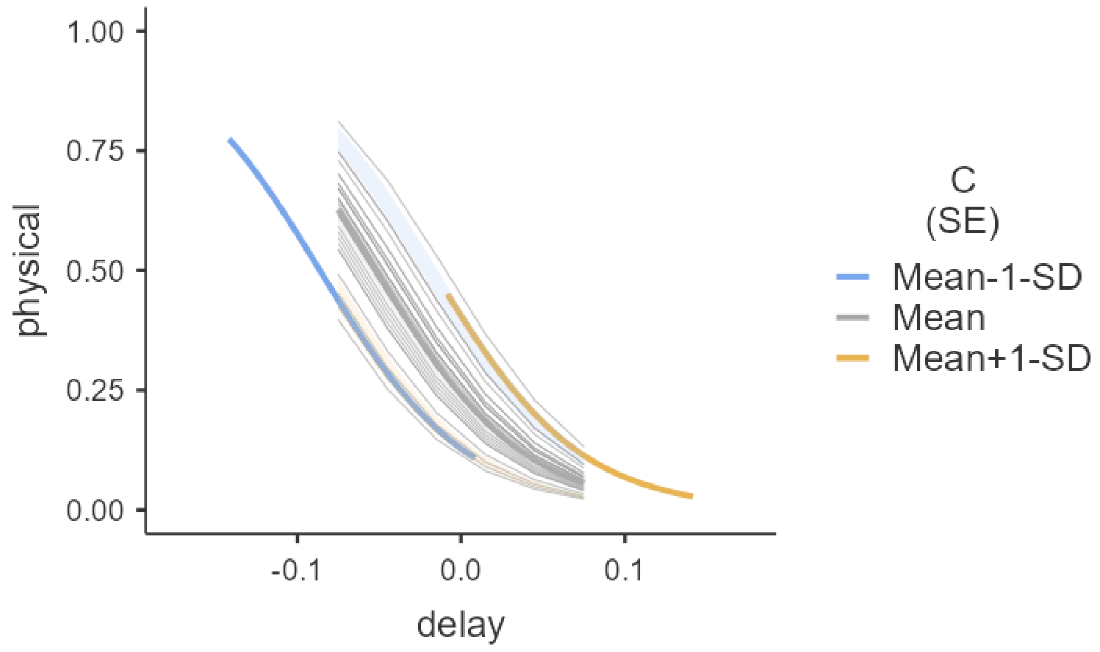

Note: Random effects are plotted by participant

Same as before but after logit transform…

n_bouncing_cycles = 1

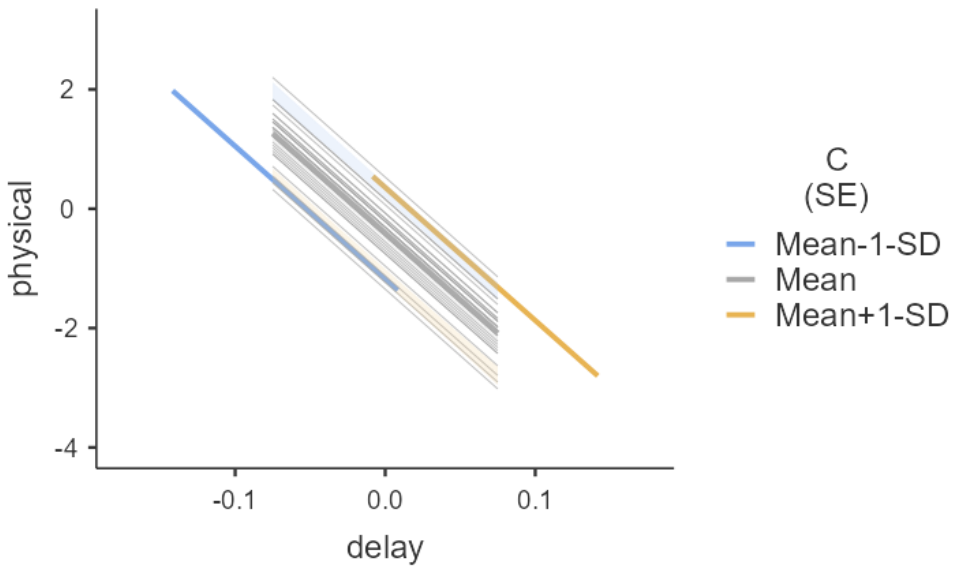

n_bouncing_cycles = 3

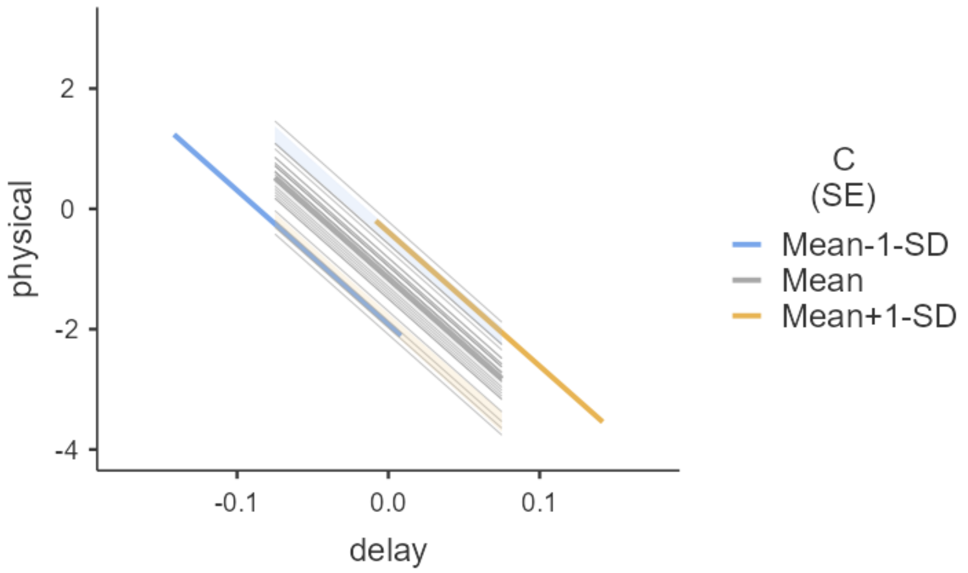

Note that although the two n_bouncing_cycles figures above may look similar, the mean values for n_bouncing_cycles = 3 are smaller than for n_bouncing_cycles = 1

APPENDIX 3

Generalized Mixed Models (Expt2), "physical" response, with n_bouncing_cycles, delay and C as factors

Info	Value	Comment
Model Type	Logistic	Model for binary y
Call	glm	**physical ~ 1 + n_bouncing_cycles + delay + C + n_bouncing_cycles:delay + n_bouncing_cycles:C + delay:C + n_bouncing_cycles:delay:C + (1 | participant)**
Link function	Logit	Log of the odd of y=1 over y=0
Direction	P(y=1)/P(y=0)	P(physical = 1) / P(physical = 0)
Distribution	Binomial	Dichotomous event distribution of y
LogLikel.	-1378.629	Unconditional Log-Likelihood
-2*LogLikel.	2757.257	Unconditional absolute deviance
Deviance	2686.686	Conditional relative deviance
**R-squared**	**0.422**	**Marginal**
**R-squared**	**0.464**	**Conditional**
AIC	2807.260	Less is better
BIC	2956.396	Less is better
Residual DF	2855.000	
Chi-squared/DF	0.934	Overdispersion indicator
Converged	yes	
Optimizer	bobyqa	

Fixed Effect Omnibus tests

	X^2	df	p
n_bouncing_cycles	51.6	1.00	< .001
delay	412.7	5.00	< .001
C	164.1	1.00	< .001
n_bouncing_cycles ✻ delay	32.7	5.00	< .001
n_bouncing_cycles ✻ C	32.6	1.00	< .001
delay ✻ C	15.5	5.00	0.008
n_bouncing_cycles ✻ delay ✻ C	13.6	5.00	0.018

Random Components

Groups	Name	SD	Variance	ICC
participant	(Intercept)	0.506	0.256	**0.0722**
Residuals		1.000	1.000	.

Note. Number of Obs: 2880 , groups: participant 30

Results Plots

n_bouncing_cycles = 1

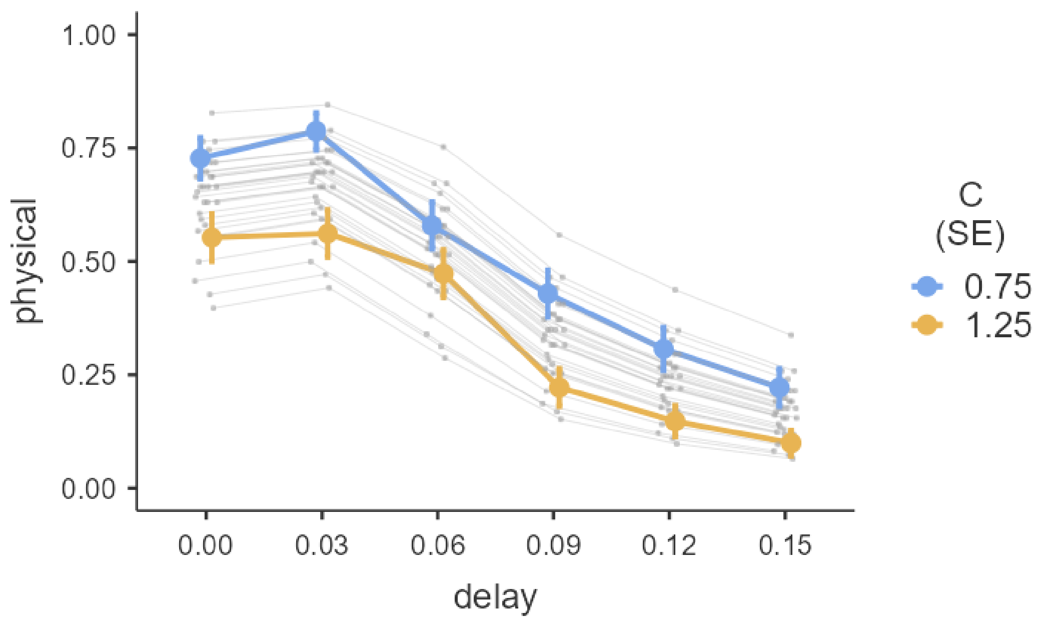

n_bouncing_cycles = 3

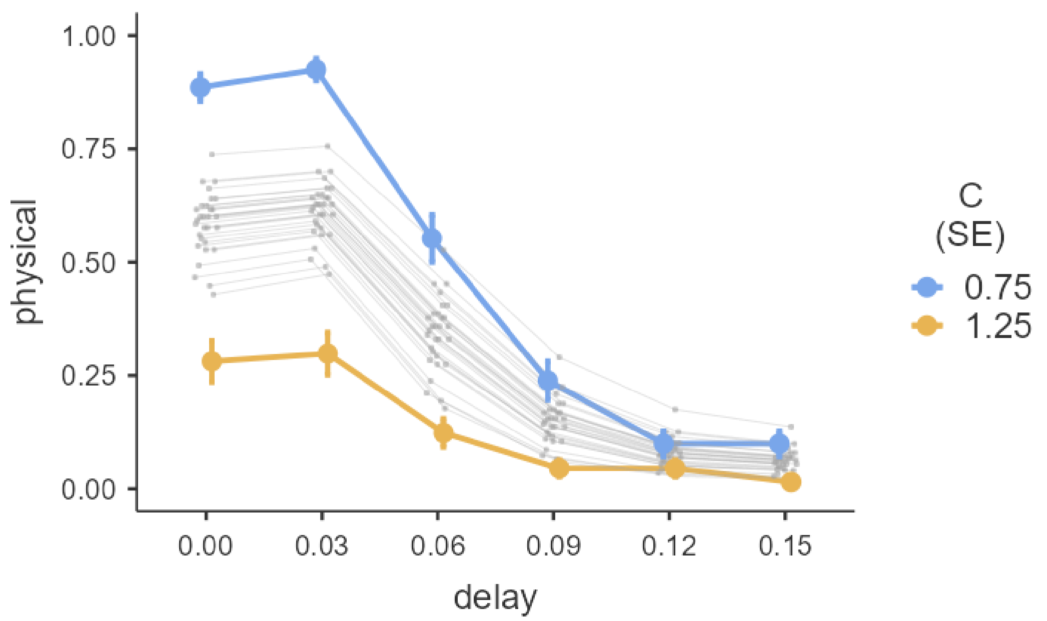

Note: Random effects are plotted by participant

APPENDIX 4

Same as in Appendix 2 but with a random slope effect for "Delay"

(see https://gamlj.github.io/mixed_example1.html#Set_up for an explanation of our to run Random slope models)

Model Info

Info	Value	Comment
Model Type	Logistic	Model for binary y
**Call**	**glm**	**physical ~ 1 + C + delay + n_bouncing_cycles + (1 + delay | participant)**
Link function	Logit	Log of the odd of y=1 over y=0
Direction	P(y=1)/P(y=0)	P(physical = 1) / P(physical = 0)
Distribution	Binomial	Dichotomous event distribution of y
LogLikel.	-1376.962	Unconditional Log-Likelihood
-2*LogLikel.	2753.923	Unconditional absolute deviance
Deviance	2591.663	Conditional relative deviance
R-squared	0.378	Marginal
R-squared	0.543	Conditional
AIC	2767.920	Less is better
BIC	2809.682	Less is better
Residual DF	2873.000	
Chi-squared/DF	0.934	Overdispersion indicator
Converged	yes	
Optimizer	bobyqa	

Note that the conditional R^2 (0.543) has increased as compared to the Random intercept model (0.398) of Appendix 2, and that the AIC and BCI have decreased (more interesting solution).

Random Components

Groups	Name	SD	Variance	ICC
participant	(Intercept)	0.559	0.313	0.0868
	delay	18.275	333.985	
Residuals		1.000	1.000	.

Note. Number of Obs: 2880 , groups: participant 30

Random Parameters correlations

Groups	Param.1	Param.2	Corr.
participant	(Intercept)	delay	0.474

The correlation between intercepts and slopes, r = 0.474, indicates more frequent "physical" judgments on average (intercept) when the effect of delay is greater.

Effects Plots

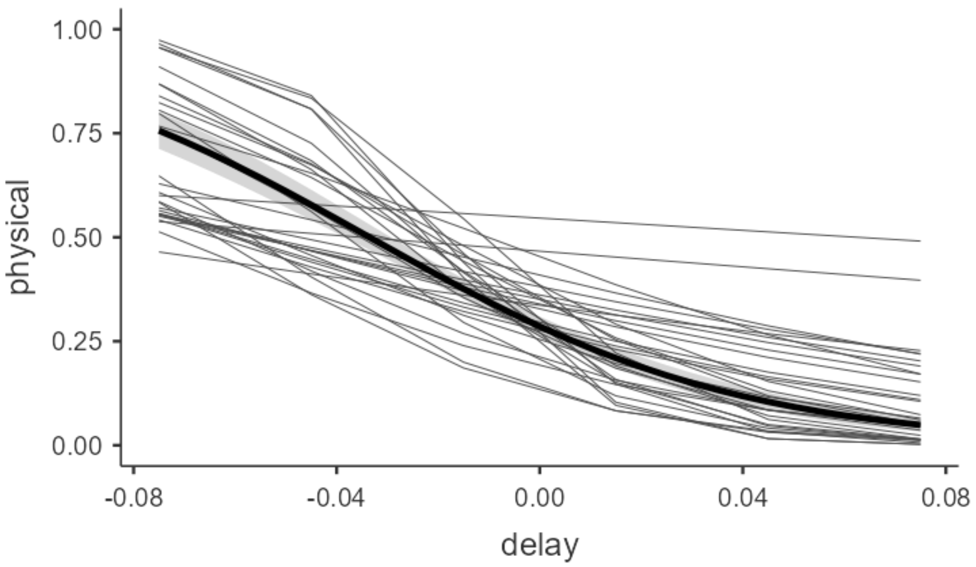

APPENDIX 5

Expt 2, *ANOVA on response proportion* with Response category (physical, animated, other), C, Delay, n_bouncing_cycles as Within Subjects factors

Cases	Sum of Squares	H-F corrected df	Mean Squares	F	*p*
response	36.253	1.708	21.228	42.077	0.0000000001
Residuals	24.986	49.527	0.505		
response ✻ n_bouncing_cycles	4.298	1.726	2.490	13.623	0.0000439829
Residuals	9.150	50.060	0.183		
response ✻ C	30.094	1.514	19.875	20.232	0.0000042513
Residuals	43.135	43.910	0.982		
response ✻ Delay	47.380	2.285	20.734	27.808	0.0000000003
Residuals	49.412	66.268	0.746		
**response ✻ n_bouncing_cycles ✻ C**	**2.612**	**1.969**	**1.326**	**4.294**	**0.0187751974**
**Residuals**	**17.638**	**57.114**	**0.309**		
response ✻ n_bouncing_cycles ✻ [illegible]	0.964	7.851	0.123	1.533	0.1482212363
Residuals	18.244	227.681	0.080		
response ✻ C ✻ Delay	4.185	7.442	0.562	6.964	0.0000000873
Residuals	17.429	215.812	0.081		
response ✻ n_bouncing_cycles ✻ C [illegible]	3.288	7.055	0.466	5.849	0.0000031341
Residuals	16.305	204.587	0.080		

Note. Sphericity corrections not available for factors with 2 levels.

Note. Type III Sum of Squares

[a] Mauchly's test of sphericity indicates that the assumption of sphericity is violated (p < .05).

Note: because the total proportion of the response category is 1 there is no variance associated with the following sources of variance, which are consequently not reported in the above Table: n_bouncing_cycles; C; Delay; n_bouncing_cycles*C; n_bouncing_cycles*Delay; C; Delay ; n_bouncing_cycles*C*Delay;

Different figures for two different interactions…

Cases	Sum of Squares	H-F corrected df	Mean Squares	F	*p*
response ✻ n_bouncing_cycles ✻ C	2.612	1.969	1.326	4.294	0.01878
Residuals	17.638	57.114	0.309		

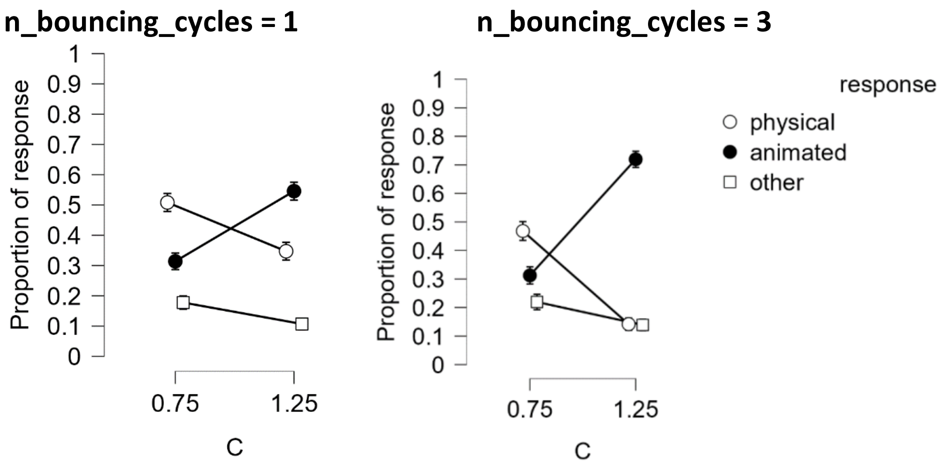

Cases	Sum of Squares	H-F corrected df	Mean Squares	F	*p*
response ✻ C ✻ Delay	4.185	7.442	0.562	6.964	0.000000087
Residuals	17.429	215.812	0.081		

Descriptives plots

C = 0.75

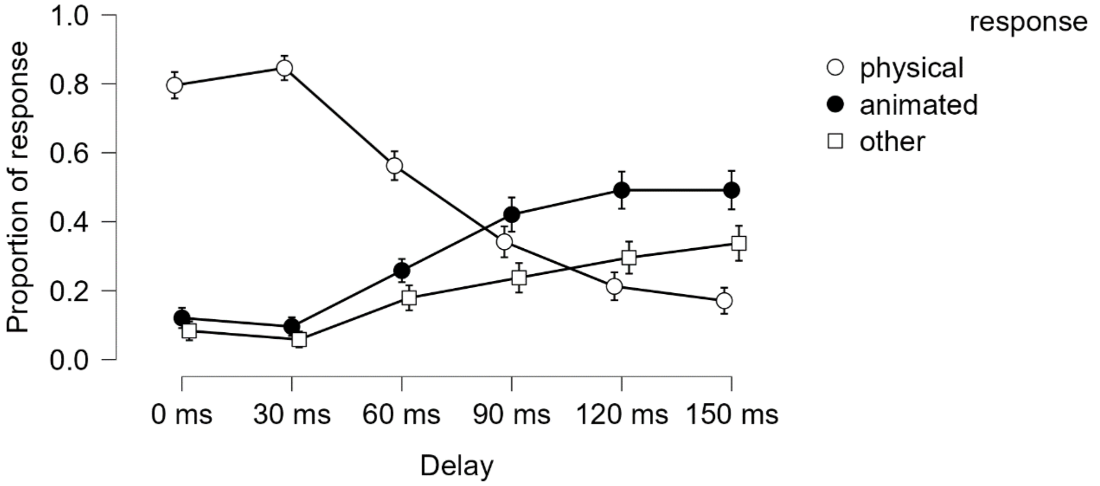

C = 1.25

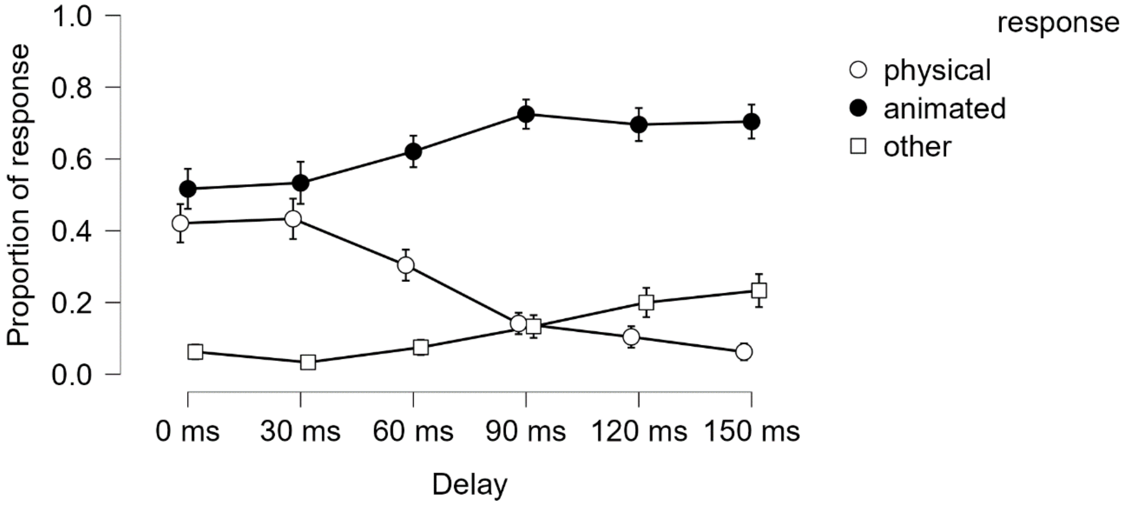

APPENDIX 6

Expt 2, *ANOVA on response proportion* with Response category (physical, animated), C, Delay, n_bouncing_cycles as Within Subjects factors

Within Subjects Effects

Cases	Sphericity Correction	Sum of Squares	df	Mean Square	F	p
response	None	4.091	1.000	4.091	17.604	2.346e-4
Residuals	None	6.739	29.000	0.232		
n_bouncing_cycles	None	0.122	1.000	0.122	2.470	0.127
Residuals	None	1.431	29.000	0.049		
C	None	0.516	1.000	0.516	4.736	0.038
Residuals	None	3.158	29.000	0.109		
Delay	None	2.748 [a]	5.000 [a]	0.550 [a]	13.679 [a]	6.146e-11 [a]
	H-F	2.748	1.684	1.632	13.679	5.080e-5
Residuals	None	5.826	145.000	0.040		
	H-F	5.826	48.827	0.119		
response ✻ n_bouncing_cycles	None	3.932	1.000	3.932	23.487	3.888e-5
Residuals	None	4.855	29.000	0.167		
response ✻ C	None	28.547	1.000	28.547	24.593	2.842e-5
Residuals	None	33.663	29.000	1.161		
n_bouncing_cycles ✻ C	None	0.002	1.000	0.002	0.029	0.865
Residuals	None	2.109	29.000	0.073		
response ✻ Delay	None	39.136 [a]	5.000 [a]	7.827 [a]	35.541 [a]	1.281e-23 [a]
	H-F	39.136	1.533	25.524	35.541	8.630e-9
Residuals	None	31.933	145.000	0.220		
	H-F	31.933	44.465	0.718		
n_bouncing_cycles ✻ Delay	None	0.153 [a]	5.000 [a]	0.031 [a]	1.869 [a]	0.103 [a]
	H-F	0.153	3.853	0.040	1.869	0.123
Residuals	None	2.379	145.000	0.016		
	H-F	2.379	111.748	0.021		
C ✻ Delay	None	0.126 [a]	5.000 [a]	0.025 [a]	1.719 [a]	0.134 [a]
	H-F	0.126	3.703	0.034	1.719	0.156
Residuals	None	2.130	145.000	0.015		
	H-F	2.130	107.391	0.020		
response ✻ n_bouncing_cycles ✻ C	None	2.605	1.000	2.605	6.679	0.015
Residuals	None	11.313	29.000	0.390		
response ✻ n_bouncing_cycles ✻ Delay	None	0.504 [a]	5.000 [a]	0.101 [a]	1.317 [a]	0.260 [a]
	H-F	0.504	4.019	0.126	1.317	0.268
Residuals	None	11.106	145.000	0.077		
	H-F	11.106	116.538	0.095		
response ✻ C ✻ Delay	None	3.806	5.000	0.761	10.000	3.079e-8
	H-F	3.806	4.217	0.903	10.000	2.924e-7
Residuals	None	11.039	145.000	0.076		
	H-F	11.039	122.297	0.090		
n_bouncing_cycles ✻ C ✻ Delay	None	0.331 [a]	5.000 [a]	0.066 [a]	4.689 [a]	5.345e-4 [a]
	H-F	0.331	3.469	0.096	4.689	0.003
Residuals	None	2.050	145.000	0.014		
	H-F	2.050	100.601	0.020		
response ✻ n_bouncing_cycles ✻ C ✻ Delay	None	2.294 [a]	5.000 [a]	0.459 [a]	6.551 [a]	1.592e-5 [a]
	H-F	2.294	3.781	0.607	6.551	1.257e-4
Residuals	None	10.155	145.000	0.070		
	H-F	10.155	109.660	0.093		

Note. Sphericity corrections not available for factors with 2 levels.

Note. Type III Sum of Squares

[a] Mauchly's test of sphericity indicates that the assumption of sphericity is violated ($p < .05$).
